# Construction and validation of nomograms combined with novel machine learning algorithms to predict early death of patients with metastatic colorectal cancer

**DOI:** 10.3389/fpubh.2022.1008137

**Published:** 2022-12-20

**Authors:** Yalong Zhang, Zunni Zhang, Liuxiang Wei, Shujing Wei

**Affiliations:** ^1^Department of Ultrasound Medicine, The Fifth Affiliated Hospital of Guangxi Medical University, Nanning, China; ^2^Department of Clinical Laboratory, The People's Hospital of Guangxi Zhuang Autonomous Region, Nanning, China

**Keywords:** metastatic colorectal cancer, dynamic nomogram, novel machine learning, early death, SEER

## Abstract

**Purpose:**

The purpose of this study was to investigate the clinical and non-clinical characteristics that may affect the early death rate of patients with metastatic colorectal carcinoma (mCRC) and develop accurate prognostic predictive models for mCRC.

**Method:**

Medical records of 35,639 patients with mCRC diagnosed from 2010 to 2019 were obtained from the SEER database. All the patients were randomly divided into a training cohort and a validation cohort in a ratio of 7:3. X-tile software was utilized to identify the optimal cutoff point for age and tumor size. Univariate and multivariate logistic regression models were used to determine the independent predictors associated with overall early death and cancer-specific early death caused by mCRC. Simultaneously, predictive and dynamic nomograms were constructed. Moreover, logistic regression, random forest, CatBoost, LightGBM, and XGBoost were used to establish machine learning (ML) models. In addition, receiver operating characteristic curves (ROCs) and calibration plots were obtained to estimate the accuracy of the models. Decision curve analysis (DCA) was employed to determine the clinical benefits of ML models.

**Results:**

The optimal cutoff points for age were 58 and 77 years and those for tumor size of 45 and 76. A total of 15 independent risk factors, namely, age, marital status, race, tumor localization, histologic type, grade, N-stage, tumor size, surgery, radiation, chemotherapy, bone metastasis, brain metastasis, liver metastasis, and lung metastasis, were significantly associated with the overall early death rate of patients with mCRC and the cancer-specific early death rate of patients with mCRC, following which nomograms were constructed. The ML models revealed that the random forest model accurately predicted outcomes, followed by logistic regression, CatBoost, XGBoost, and LightGBM models. Compared with other algorithms, the random forest model provided more clinical benefits than other models and can be used to make clinical decisions in overall early death and specific early death caused by mCRC.

**Conclusion:**

ML algorithms combined with nomograms may play an important role in distinguishing early deaths owing to mCRC and potentially help clinicians make clinical decisions and follow-up strategies.

## 1. Introduction

Colorectal carcinoma (CRC) is an aggressive malignant tumor and the third most common malignancy. It is the fourth leading contributor to cancer-related deaths in the world. In 2020, there were more than 1.1 million new CRC cases, and about 570,000 deaths were caused by CRC (1). Research has revealed that by 2030, the number of newly diagnosed patients with CRC is expected to increase to more than 2.2 million and the number of deaths to 1.1 million (2). Distant metastasis is the main leading cause of poor prognosis in patients with CRC, and about 25% of patients with CRC have been found to have distant metastasis at initial diagnosis (3). The risk of developing CRC depends on lifestyle, behavioral characteristics, and genetic factors. With the widespread knowledge of physical examination, the availability of treatments (surgical resection, chemotherapy, radiotherapy, and immunotherapy), and the discovery of early biomarkers, the prognosis of patients with CRC has improved significantly (4, 5). However, the prognosis of patients with mCRC is strikingly poor: The 5-year survival rate is only 10%, and the median survival time is about 5 months (6). Therefore, it is of great importance to identify risk factors of early death in patients with mCRC.

Clinical and pathological variables such as age, sex, race, and tumor size have been recognized as risk factors for cancer (7). The nomogram was found to be an advanced approach capable of predicting individual oncologic prognosis based on comprehensive characteristics (8). Moreover, as an emerging intersectional method, ML is adept at relating multiple variables and accurately predicting outcomes (9). Therefore, multiple ML predictive models have recently been used in disease diagnosis, prognostic prediction, and clinical decision-making (10, 11).

The purpose of this research was not only to use nomograms to evaluate the factors contributing to early death in patients with mCRC but also to find an approach with higher precision and clinical applicability for predicting early death in patients with mCRC based on machine learning algorithms, which could potentially help clinicians make clinical decisions and follow-up strategies.

## 2. Materials and methods

### 2.1. Patient cohorts

Surveillance, Epidemiology, and End Results (SEER, https://seer.cancer.gov/) is the National Cancer Institute's open public database that contains cancer incidence and survival data of 17 established cancer registries across the United States and accounts for approximately 26.5% of incidence and survival rates of patients with cancer (12). In this study, SEER^*^Stat software (version 8.4.0) was used to extract clinical data of patients with mCRC from 2010 to 2019 (reference number 11788-Nov2021). The inclusion criteria for patients with mCRC in this investigation were as follows: (1) patients with tumor location codes of C18.0, C18.2–18.7, C19.9, and C20.9; (2) patients confirmed with stage IV CRC by histopathology; (3) patients aged 18–99 years old; (4) patients with only one primary site; and (5) patients with complete information on survival status. Patients diagnosed only by autopsy were excluded.

**Figure 1:**
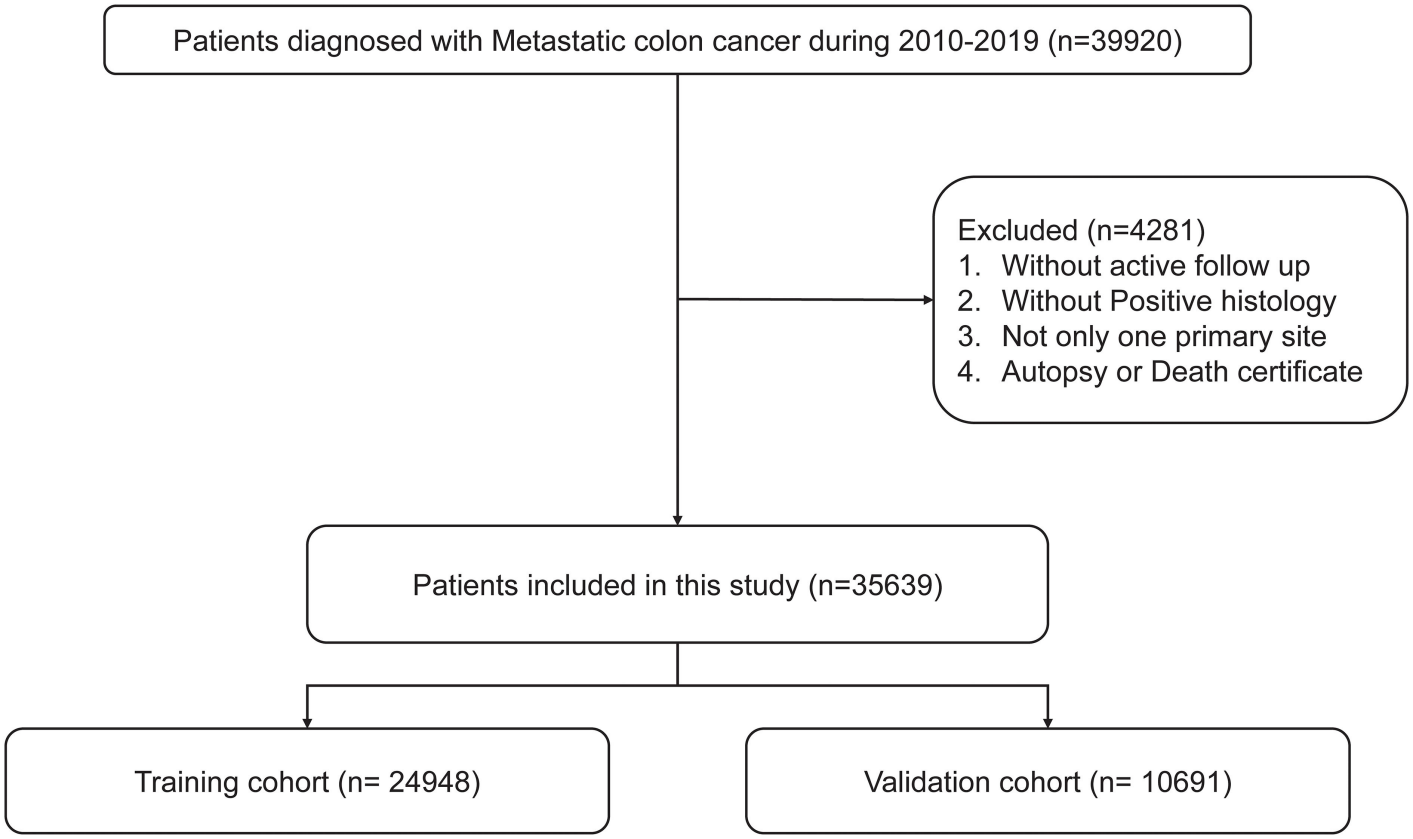
Flowchart for selection procedure of patients with mCRC.

The screening process is shown in Figure 1. According to previous studies, early death was defined as death of patients within 3 months of diagnosis (13–15). All the included patients with mCRC were divided into a training cohort (accounting for 70%) and a validation cohort (accounting for 30%). X-tile was used to calculate the optimal cutoff point of patients' age and tumor size (16).

### 2.2. Construction of nomograms and novel machine learning

We compared the characteristics of the training and validation groups and analyzed the factors to predict early death in patients using univariate logistic analysis. Subsequently, significant variables were evaluated using stepwise multivariate logistic regression analysis, and the independent predictors associated with early death in patients were determined. According to the nomogram, the probability of early death in patients can be calculated. Moreover, the likelihood of early death in patients can be estimated by using the dynamic nomograms. To ensure the stability of the model, 10-fold cross-validation was used to evaluate the predictive ability of the model. Our model was then repeatedly tested and tuned, and the parameters to obtain the optimal model were determined. Independent predictors were included in five ML algorithms, and AUC was calculated to identify the top performing ML model. The differences between AUCs were compared by a bootstrap test. Calibration plots and DCA were used to assess calibration capability and clinical benefits, respectively.

### 2.3. Statistical analysis

Demographic and clinical factors were described by numbers and percentages. A pie chart was used to show the overall distribution of the data in the study. Pearson's chi-square test was used to evaluate the clinicopathological variables between the training and validation cohorts. Variables with a *P* < 0.05 in the univariate logistic analysis were screened for multivariate stepwise logistic regression to identify the possible independent risk factors. In addition, multicollinearity diagnostics in statistical modeling was performed by evaluating correlations, variance inflation factors, and eigenvalues. The forest plot obtained by using the R package “forestplot” showed the multivariate logistic analysis results of overall early death and cancer-specific early death, respectively. Nomograms were constructed from the results of univariate and multivariate analyses using the “rms” package. Simultaneously, a more flexible and better visualized dynamic nomogram was obtained through the “DynNom” package. In this study, three newly developed gradient boosting models (GBMs), namely, CatBoost, LightGBM, and XGBoost, and random forest and logistic regression models were implemented by “CatBoost,” “LightGBM,” “XGBoost,” “random forest,” and “rms” packages, respectively. The “pROC,” “rms,” and “rmda” packages were used to generate ROC, calibration, and DCA curves, respectively (https://github.com/mdbrown/rmda). All statistical analyses were performed using R software (version 4.2.1, http://www.r-project.org/).

## 3. Results

### 3.1. Demographic and clinical characteristics

**Figure 2:**
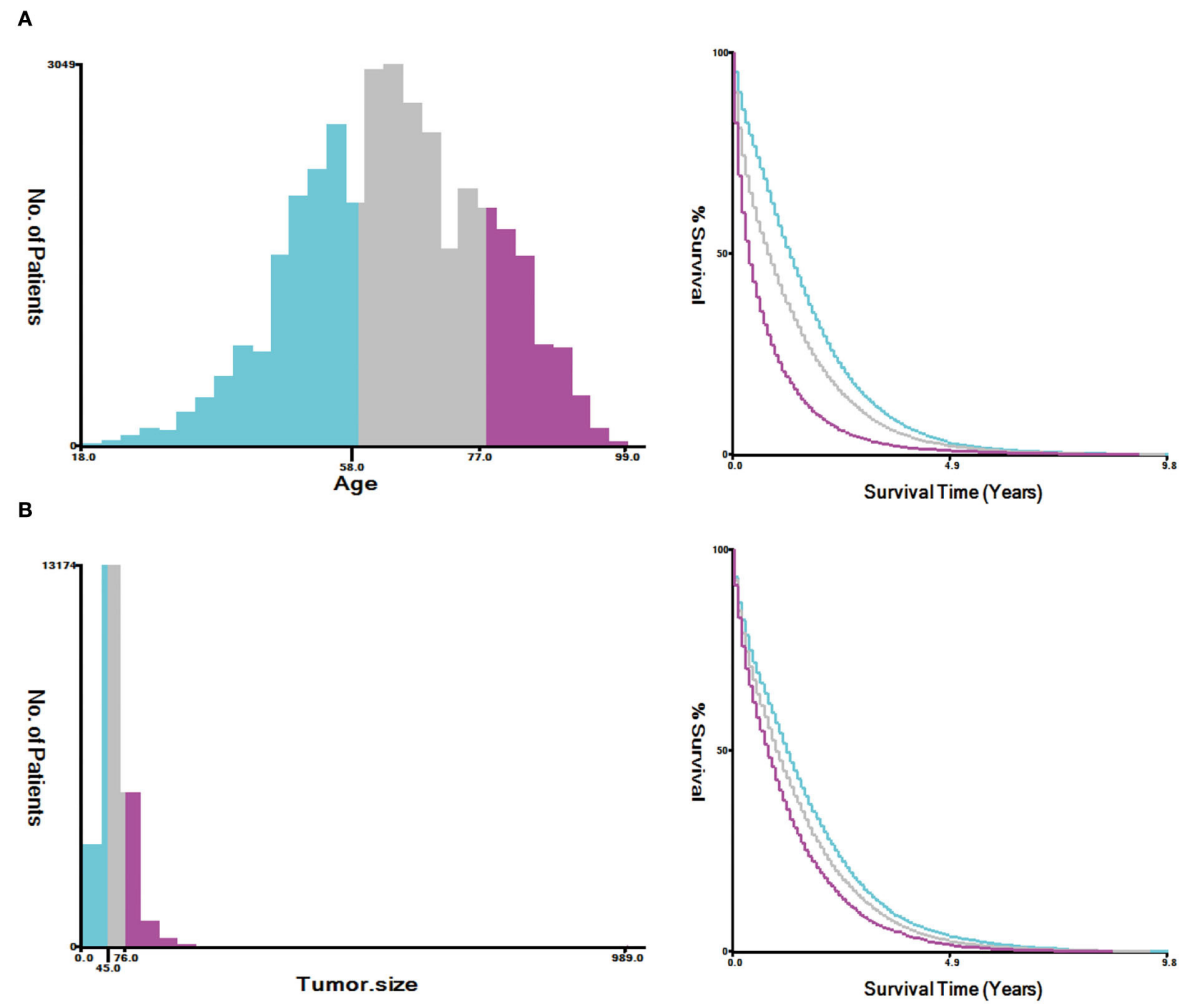
Estimation of the appropriate cutoff value for age **(A)** and tumor size **(B)** by X-tile analysis.

A total of 35,639 patients with mCRC were included in this study, and the patients were randomly divided into the training cohort (*n* = 24,948) and validation cohort (*n* = 10,691). The results analyzed by X-tile software revealed that the optimal cutoff point for age was 58 and 77 years, and the optimal cutoff point for tumor size was 45 and 76 (Figure 2). The data distribution was displayed by pie chart (Supplementary Figure S1).

As shown in Table 1, 29.2% (10,396/35,639) of patients with mCRC have died within 3 months after diagnosis, and 26.8% (9,535/35,639) of the patients died of cancer. Most of the patients with mCRC were white (75.3%) and with household incomes of $55,000–$69,999 (39.6%) and >$70,000 (33.1%), and liver metastasis was the most common type (75%) when compared with bone (6.7%), brain (1.5%), and lung (26.7%) metastases. Only few of the patients with mCRC received radiation therapy (11.7%), whereas many of the patients preferred chemotherapy (63.3%). The tumor was more commonly located in the right colon (34.3%) than in the left colon (28.2%), transverse colon (6.3%), rectosigmoid (9.5%), and rectum (21.8%). The early death rate was higher in white people (76.2%) than in other ethnic groups and was higher in the right colon (39.2%) than in the left colon (26.1%), transverse colon (7.6%), rectosigmoid (9.1%), and rectum (17.9%). Treatments including surgery, radiation, and chemotherapy significantly reduced early death in patients with mCRC.

**Table 1:** Demographic information of patients with mCRC.

	**Level**	**Overall**	**No early death**	**Total early death**	**Cancer-specific early death**
n		35,639	25,243	10,396	9,535
Age (%)	≤ 58	12,455 (34.9)	10,258 (40.6)	2,197 (21.1)	2,059 (21.6)
	59–77	16,479 (46.2)	11,427 (45.3)	5,052 (48.6)	4,628 (48.5)
	>77	6,705 (18.8)	3,558 (14.1)	3,147 (30.3)	2,848 (29.9)
Sex (%)	Male	19,677 (55.2)	14,092 (55.8)	5,585 (53.7)	5,098 (53.5)
	Female	15,962 (44.8)	11,151 (44.2)	4,811 (46.3)	4,437 (46.5)
Marital status (%)	Married	17,116 (48.0)	12,938 (51.3)	4,178 (40.2)	3,850 (40.4)
	Unmarried	16,524 (46.4)	10,960 (43.4)	5,564 (53.5)	5,099 (53.5)
	Unknown	1,999 (5.6)	1,345 (5.3)	654 (6.3)	586 (6.1)
Race (%)	White	26,830 (75.3)	18,908 (74.9)	7,922 (76.2)	7,277 (76.3)
	Black	5,264 (14.8)	3,712 (14.7)	1,552 (14.9)	1,420 (14.9)
	Other	3,464 (9.7)	2,581 (10.2)	883 (8.5)	807 (8.5)
	Unknown	81 (0.2)	42 (0.2)	39 (0.4)	31 (0.3)
Median household income (%)	<$40,000	2,139 (6.0)	1,510 (6.0)	629 (6.1)	579 (6.1)
	$40,000–$54,999	7,568 (21.2)	5,300 (21.0)	2,268 (21.8)	2,096 (22.0)
	$55,000–$69,999	14,128 (39.6)	9,967 (39.5)	4,161 (40.0)	3,820 (40.1)
	>$70,000	11,803 (33.1)	8,465 (33.5)	3,338 (32.1)	3,040 (31.9)
	Unknown	1 (0.0)	1 (0.0)	0 (0.0)	0 (0.0)
Tumor localization (%)	Right colon	12,209 (34.3)	8,133 (32.2)	4,076 (39.2)	3,728 (39.1)
	Transverse	2,240 (6.3)	1,445 (5.7)	795 (7.6)	753 (7.9)
	Left colon	10,039 (28.2)	7,329 (29.0)	2,710 (26.1)	2,486 (26.1)
	Rectosigmoid	3,391 (9.5)	2,442 (9.7)	949 (9.1)	877 (9.2)
	Rectum	7,760 (21.8)	5,894 (23.3)	1,866 (17.9)	1,691 (17.7)
Histologic type (%)	Adenocarcinoma	32,994 (92.6)	23,770 (94.2)	9,224 (88.7)	8,457 (88.7)
	Not adenocarcinoma	2,645 (7.4)	1,473 (5.8)	1,172 (11.3)	1,078 (11.3)
Grade (%)	I–II	17,583 (49.3)	13,916 (55.1)	3,667 (35.3)	3,327 (34.9)
	III–IV	8,207 (23.0)	5,507 (21.8)	2,700 (26.0)	2,477 (26.0)
	Unknown	9,849 (27.6)	5,820 (23.1)	4,029 (38.8)	3,731 (39.1)
T (%)	T1-T2	3,746 (10.5)	2,581 (10.2)	1,165 (11.2)	1,070 (11.2)
	T3-T4	20,261 (56.9)	15,668 (62.1)	4,593 (44.2)	4,196 (44.0)
	Unknown	11,632 (32.6)	6,994 (27.7)	4,638 (44.6)	4,269 (44.8)
N (%)	N0-N1	21,149 (59.3)	15,263 (60.5)	5,886 (56.6)	5,384 (56.5)
	N2	8,582 (24.1)	6,656 (26.4)	1,926 (18.5)	1,771 (18.6)
	Unknown	5,908 (16.6)	3,324 (13.2)	2,584 (24.9)	2,380 (25.0)
Tumor size (%)	≤ 45	8,455 (23.7)	6,650 (26.3)	1,805 (17.4)	1,633 (17.1)
	46–76	10,293 (28.9)	7,691 (30.5)	2,602 (25.0)	2,390 (25.1)
	>76	4,608 (12.9)	3,235 (12.8)	1,373 (13.2)	1,260 (13.2)
	Unknown	12,283 (34.5)	7,667 (30.4)	4,6g6g616 (44.4)	4,252 (44.6)
Surgery (%)	Yes	16,420 (46.1)	13,116 (52.0)	3,304 (31.8)	2,985 (31.3)
	No	19,141 (53.7)	12,073 (47.8)	7,068 (68.0)	6,526 (68.4)
	Unknown	78 (0.2)	54 (0.2)	24 (0.2)	24 (0.3)
Non primary surgery (%)	Yes	5,069 (14.2)	4,138 (16.4)	931 (9.0)	847 (8.9)
	No	30,464 (85.5)	21,028 (83.3)	9,436 (90.8)	8,661 (90.8)
	Unknown	106 (0.3)	77 (0.3)	29 (0.3)	27 (0.3)
Radiation (%)	Yes	4,180 (11.7)	3,534 (14.0)	646 (6.2)	607 (6.4)
	No/Unknown	31,459 (88.3)	21,709 (86.0)	9,750 (93.8)	8,928 (93.6)
Chemotherapy (%)	Yes	22,559 (63.3)	20,051 (79.4)	2,508 (24.1)	2,336 (24.5)
	No/Unknown	13,080 (36.7)	5,192 (20.6)	7,888 (75.9)	7,199 (75.5)
Bone metastasis (%)	No	32,120 (90.1)	23,154 (91.7)	8,966 (86.2)	8,207 (86.1)
	Yes	2,380 (6.7)	1,373 (5.4)	1,007 (9.7)	951 (10.0)
	Unknown	1,139 (3.2)	716 (2.8)	423 (4.1)	377 (4.0)
Brain metastasis (%)	No	33,862 (95.0)	24,210 (95.9)	9,652 (92.8)	8,856 (92.9)
	Yes	552 (1.5)	271 (1.1)	281 (2.7)	265 (2.8)
	Unknown	1,225 (3.4)	762 (3.0)	463 (4.5)	414 (4.3)
Liver metastasis (%)	No	8,384 (23.5)	6,217 (24.6)	2,167 (20.8)	1,938 (20.3)
	Yes	26,742 (75.0)	18,675 (74.0)	8,067 (77.6)	7,451 (78.1)
	Unknown	513 (1.4)	351 (1.4)	162 (1.6)	146 (1.5)
Lung metastasis (%)	No	24,917 (69.9)	18,041 (71.5)	6,876 (66.1)	6,276 (65.8)
	Yes	9,530 (26.7)	6,456 (25.6)	3,074 (29.6)	2,855 (29.9)
	Unknown	1,192 (3.3)	746 (3.0)	446 (4.3)	404 (4.2)

There were no significant differences in age, sex, marital status, race, median household income, tumor localization, histologic type, grade-stage, TN-stage (AJCC 8th version), tumor size, surgery, radiotherapy, chemotherapy, non-primary surgery, bone metastasis, brain metastasis, liver metastasis, and lung metastasis between the training and testing cohorts, with all *p* > 0.05 (Table 2). Therefore, the training and validation cohorts could be used for the follow-up research.

**Table 2:** Demographic information of patients with mCRC in training and validation cohorts.

	**Level**	**Overall**	**Training cohort**	**Validation cohort**	***p***
*n*		35,639	24,948	10,691	
Age (%)	≤ 58	12,455 (34.9)	8,694 (34.8)	3,761 (35.2)	0.72
	59–77	16,479 (46.2)	1,1536 (46.2)	4,943 (46.2)	
	>77	6,705 (18.8)	4,718 (18.9)	1,987 (18.6)	
Sex (%)	Male	19,677 (55.2)	13,782 (55.2)	5,895 (55.1)	0.867
	Female	15,962 (44.8)	11,166 (44.8)	4,796 (44.9)	
Marital status (%)	Married	17,116 (48.0)	12,038 (48.3)	5,078 (47.5)	0.413
	Unmarried	16,524 (46.4)	11,512 (46.1)	5,012 (46.9)	
	Unknown	1,999 (5.6)	1,398 (5.6)	601 (5.6)	
Race (%)	White	26,830 (75.3)	18,745 (75.1)	8,085 (75.6)	0.373
	Black	5,264 (14.8)	3,710 (14.9)	1,554 (14.5)	
	Other	3,464 (9.7)	2,442 (9.8)	1,022 (9.6)	
	Unknown	81 (0.2)	51 (0.2)	30 (0.3)	
Median household income (%)	<$40,000	2,139 (6.0)	1,533 (6.1)	606 (5.7)	0.156
	$40,000–$54,999	7,568 (21.2)	5,330 (21.4)	2,238 (20.9)	
	$55,000–$69,999	14,128 (39.6)	9,854 (39.5)	4,274 (40.0)	
	>$70,000	11,803 (33.1)	8,231 (33.0)	3,572 (33.4)	
	Unknown	1 (0.0)	0 (0.0)	1 (0.0)	
Tumor localization (%)	Right colon	12,209 (34.3)	8,578 (34.4)	3,631 (34.0)	0.26
	Transverse	2,240 (6.3)	1,570 (6.3)	670 (6.3)	
	Left colon	10,039 (28.2)	7,053 (28.3)	2,986 (27.9)	
	Rectosigmoid	3,391 (9.5)	2,395 (9.6)	996 (9.3)	
	Rectum	7,760 (21.8)	5,352 (21.5)	2,408 (22.5)	
Histologic type (%)	Adenocarcinoma	32,994 (92.6)	23,104 (92.6)	9,890 (92.5)	0.756
	Not adenocarcinoma	2,645 (7.4)	1,844 (7.4)	801 (7.5)	
Grade (%)	I–II	17,583 (49.3)	12,321 (49.4)	5,262 (49.2)	0.895
	III–IV	8,207 (23.0)	5,728 (23.0)	2,479 (23.2)	
	Unknown	9,849 (27.6)	6,899 (27.7)	2,950 (27.6)	
T (%)	T1-T2	3,746 (10.5)	2,657 (10.7)	1,089 (10.2)	0.33
	T3-T4	20,261 (56.9)	14,133 (56.6)	6,128 (57.3)	
	Unknown	11,632 (32.6)	8,158 (32.7)	3,474 (32.5)	
N (%)	N0-N1	21,149 (59.3)	14,831 (59.4)	6,318 (59.1)	0.772
	N2	8,582 (24.1)	5,982 (24.0)	2,600 (24.3)	
	Unknown	5,908 (16.6)	4,135 (16.6)	1,773 (16.6)	
Tumor size (%)	≤ 45	8,455 (23.7)	5,878 (23.6)	2,577 (24.1)	0.738
	46–76	10,293 (28.9)	7,225 (29.0)	3,068 (28.7)	
	>76	4,608 (12.9)	3,227 (12.9)	1,381 (12.9)	
	Unknown	12,283 (34.5)	8,618 (34.5)	3,665 (34.3)	
Surgery (%)	Yes	16,420 (46.1)	11,442 (45.9)	4,978 (46.6)	0.351
	No	19,141 (53.7)	13,448 (53.9)	5,693 (53.3)	
	Unknown	78 (0.2)	58 (0.2)	20 (0.2)	
Non primary surgery (%)	Yes	5,069 (14.2)	3,507 (14.1)	1,562 (14.6)	0.387
	No	30,464 (85.5)	21,366 (85.6)	9,098 (85.1)	
	Unknown	106 (0.3)	75 (0.3)	31 (0.3)	
Radiation (%)	Yes	4,180 (11.7)	2,883 (11.6)	1,297 (12.1)	0.126
	No/Unknown	31,459 (88.3)	22,065 (88.4)	9,394 (87.9)	
Chemotherapy (%)	Yes	22,559 (63.3)	15,792 (63.3)	6,767 (63.3)	1
	No/Unknown	13,080 (36.7)	9,156 (36.7)	3,924 (36.7)	
Bone metastasis (%)	No	32,120 (90.1)	22,477 (90.1)	9,643 (90.2)	0.487
	Yes	2,380 (6.7)	1,686 (6.8)	694 (6.5)	
	Unknown	1,139 (3.2)	785 (3.1)	354 (3.3)	
Brain metastasis (%)	No	33,862 (95.0)	23,708 (95.0)	10,154 (95.0)	0.536
	Yes	552 (1.5)	395 (1.6)	157 (1.5)	
	Unknown	1,225 (3.4)	845 (3.4)	380 (3.6)	
Liver metastasis (%)	No	8,384 (23.5)	5,838 (23.4)	2,546 (23.8)	0.691
	Yes	26,742 (75.0)	18,752 (75.2)	7,990 (74.7)	
	Unknown	513 (1.4)	358 (1.4)	155 (1.4)	
Lung metastasis (%)	No	24,917 (69.9)	17,395 (69.7)	7,522 (70.4)	0.059
	Yes	9,530 (26.7)	6,744 (27.0)	2,786 (26.1)	
	Unknown	1,192 (3.3)	809 (3.2)	383 (3.6)	

### 3.2. Logistic regression analysis

**Figure 3:**
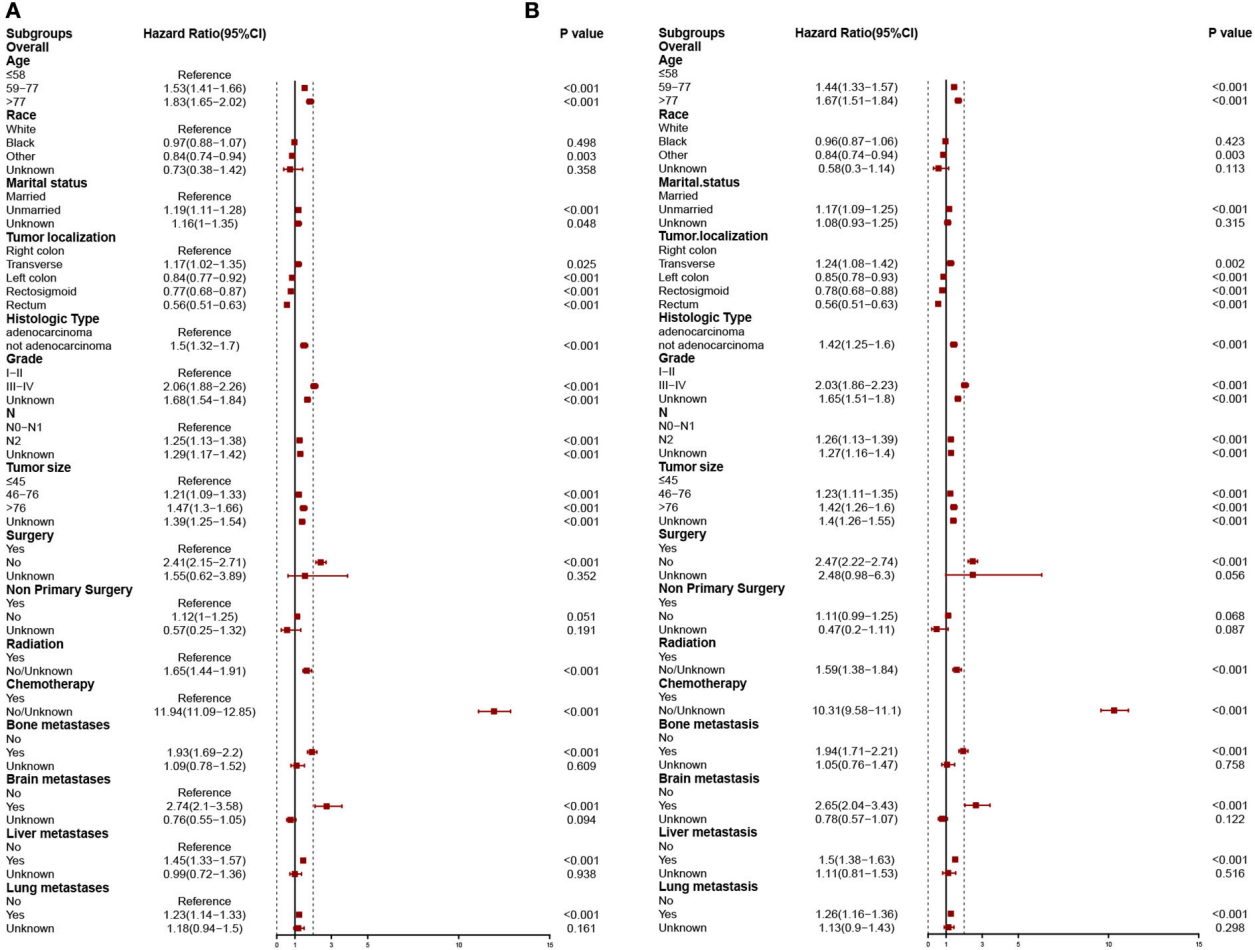
Independent predictors of stepwise logistic regression models predict overall early death **(A)** and cancer-specific early death **(B)**.

In the training cohort, the risk factors linked to the overall early death and cancer-specific early death of patients with mCRC were analyzed using univariate and multivariate logistic regression analyses. Univariate logistic analysis revealed that age at diagnosis, marital status, race, tumor localization, histologic type, grade, T-stage, N-stage, tumor size, surgery, non-primary surgery, radiation therapy, chemotherapy, bone, brain, liver, and lung metastasis were all associated with overall early death and cancer-specific early death of patients with mCRC all *p* < 0.05 (Table 3). The significant factors found by the univariate logistic analysis were included in the stepwise multivariate logistic regression, and the results illustrated that general characteristics (age, marital status, and race), tumor localization, histologic type, grade, N-stage, tumor size, and treatments (surgery, radiation, and chemotherapy), and metastases (bone, brain, liver, and lung) were independent risk factors of overall early death and cancer-specific early death of patients with mCRC, with all *p* < 0.05. The results of multivariate logistic regression were shown by forest plot (Figure 3). The results of multicollinearity diagnostic tests (pairwise correlations, variance inflation factors plot, and eigenvalues plot) revealed that there were no severe multicollinearity issues (Supplementary Figures S2, S3).

**Table 3:** Univariate logistic analysis of overall early death and cancer-specific early death in patients with mCRC.

	**Overall early death**			**Cancer-specific early death**		
	**OR**	**95% CI**	***P***	**OR**	**95% CI**	***P***
**Age**						
≤ 58	Ref			Ref		
59–77	2.07	1.93–2.21	<0.001	1.97	1.84–2.12	<0.001
>77	4.18	3.86–4.53	<0.001	3.8	3.5–4.12	<0.001
**Sex**						
Male	Ref			Ref		
Female	1.02	0.94–1.1	0.671	1.01	0.94–1.1	0.741
**Marital status**						
Married	Ref			Ref		
Unmarried	0.86	0.78–0.94	0.001	0.85	0.77–0.94	0.001
Unknown	1.53	0.87–2.69	0.137	1.22	0.67–2.21	0.51
**Race**						
White	Ref			Ref		
Black	1.08	1.03–1.14	0.004	1.1	1.04–1.16	0.001
Other	1.56	1.47–1.65	<0.001	1.52	1.43–1.61	<0.001
Unknown	1.52	1.35–1.71	<0.001	1.42	1.26–1.61	<0.001
**Median household income**						
<$40,000	Ref			Ref		
$40,000–$54,999	1.04	0.92–1.17	0.568	1.05	0.93–1.19	0.427
$55,000–$69,999	0.96	0.86–1.08	0.544	0.97	0.86–1.09	0.588
>$70,000	0.93	0.83–1.05	0.248	0.92	0.81–1.04	0.17
**Tumor localization**						
Right colon	Ref			Ref		
Transverse	1.15	1.02–1.28	0.017	1.19	1.07–1.34	0.002
Left colon	0.74	0.69–0.79	<0.001	0.75	0.69–0.8	<0.001
Rectosigmoid	0.76	0.69–0.84	<0.001	0.77	0.7–0.85	<0.001
Rectum	0.61	0.57–0.66	<0.001	0.61	0.57–0.66	<0.001
**Histologic type**						
Adenocarcinoma	Ref			Ref		
Not adenocarcinoma	2.03	1.84–2.23	<0.001	1.95	1.77–2.15	<0.001
**Grade**						
I–II	Ref			Ref		
III–IV	1.89	1.76–2.03	<0.001	1.9	1.77–2.04	<0.001
Unknown	2.65	2.49–2.83	<0.001	2.63	2.46–2.81	<0.001
**T**						
T1-T2	Ref			Ref		
T3-T4	0.65	0.6–0.72	<0.001	0.66	0.6–0.73	<0.001
Unknown	1.46	1.33–1.6	<0.001	1.44	1.31–1.59	<0.001
*N*						
N0-N1	Ref			Ref		
N2	0.75	0.7–0.81	<0.001	0.77	0.71–0.82	<0.001
Unknown	2.07	1.93–2.22	<0.001	2.02	1.88–2.17	<0.001
**Tumor size**						
≤ 45	Ref			Ref		
46–76	2.31	2.18–2.45	<0.001	2.3	2.17–2.44	<0.001
>76	2.06	1.2–3.56	0.009	2.33	1.35–4.01	0.002
Unknown	1.98	1.81–2.16	<0.001	1.96	1.78–2.15	<0.001
**Surgery**						
Yes	Ref			Ref		
No	2.06	1.26–3.37	0.004	2.02	1.22–3.35	0.006
Unknown	2.44	2.2–2.7	<0.001	2.33	2.09–2.59	<0.001
**Non primary surgery**						
Yes	Ref			Ref		
No	12.35	11.58–13.18	<0.001	10.81	10.12–11.54	<0.001
Unknown	1.83	1.66–2.03	<0.001	1.88	1.7–2.09	<0.001
**Radiation**						
Yes	Ref			Ref		
No/Unknown	1.59	1.37–1.84	<0.001	1.52	1.31–1.76	<0.001
**Chemotherapy**						
Yes	Ref			Ref		
No/Unknown	2.6	2.13–3.17	<0.001	2.61	2.14–3.19	<0.001
**Bone metastasis**						
No	Ref			Ref		
Yes	1.5	1.31–1.73	<0.001	1.44	1.25–1.67	<0.001
Unknown	1.21	1.13–1.29	<0.001	1.25	1.17–1.34	<0.001
**Brain metastasis**						
No	Ref			Ref		
Yes	1.3	1.03–1.64	0.026	1.34	1.06–1.69	0.015
Unknown	1.23	1.16–1.31	<0.001	1.26	1.18–1.34	<0.001
**Liver metastasis**						
No	Ref			Ref		
Yes	1.69	1.46–1.95	<0.001	1.61	1.39–1.87	<0.001
Unknown	1.22	1.13–1.33	<0.001	1.25	1.15–1.36	<0.001
**Lung metastasis**						
No	Ref			Ref		
Yes	1.55	1.4–1.7	<0.001	1.54	1.39–1.7	<0.001
Unknown	2.2	2.04–2.38	<0.001	2.19	2.03–2.37	<0.001

### 3.3. Dynamic nomogram construction

**Figure 4:**
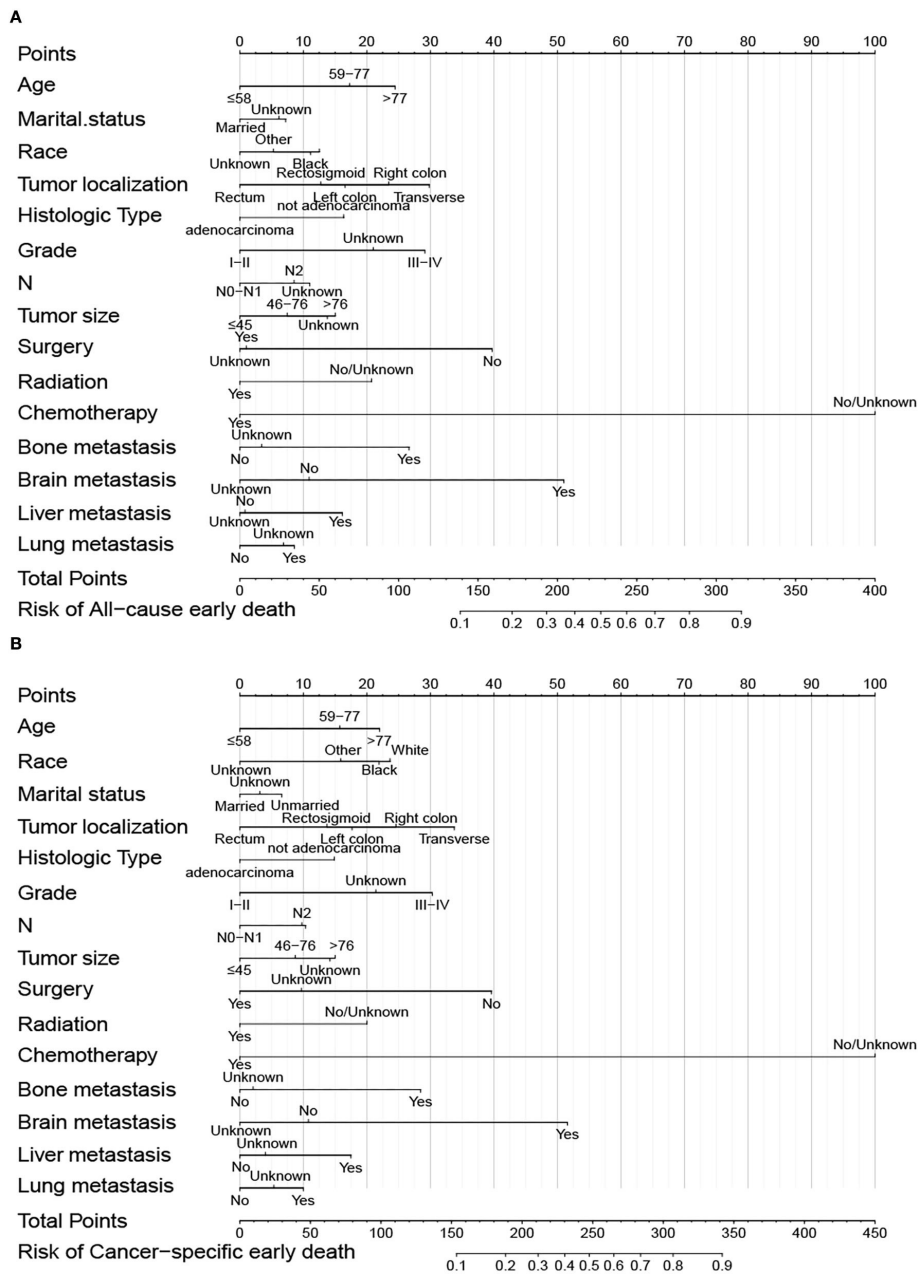
Nomogram of overall early death **(A)** and cancer-specific early death **(B)** of patients with mCRC.

Predictive nomograms were constructed according to the results of the stepwise multivariate logistic regression analysis. In the nomogram prediction models, chemotherapy had the greatest predictive value, followed by brain metastases, surgery, tumor localization, grade, and bone metastases in overall early death and cancer-specific early death (Figure 4). The odds of early death in patients with mCRC can be predicted by calculating the scores of each factor. Dynamic nomograms for total early death that could assist researchers and clinicians can be accessed at https://xiaoz7474.shinyapps.io/DynNomapp_all_cause_early_death/, and those for cancer-specific early death can be accessed at https://xiaoz7474.shinyapps.io/DynNomapp_cancer_specific_early_death/.

### 3.4. Novel machine learning algorithm

**Figure 5:**
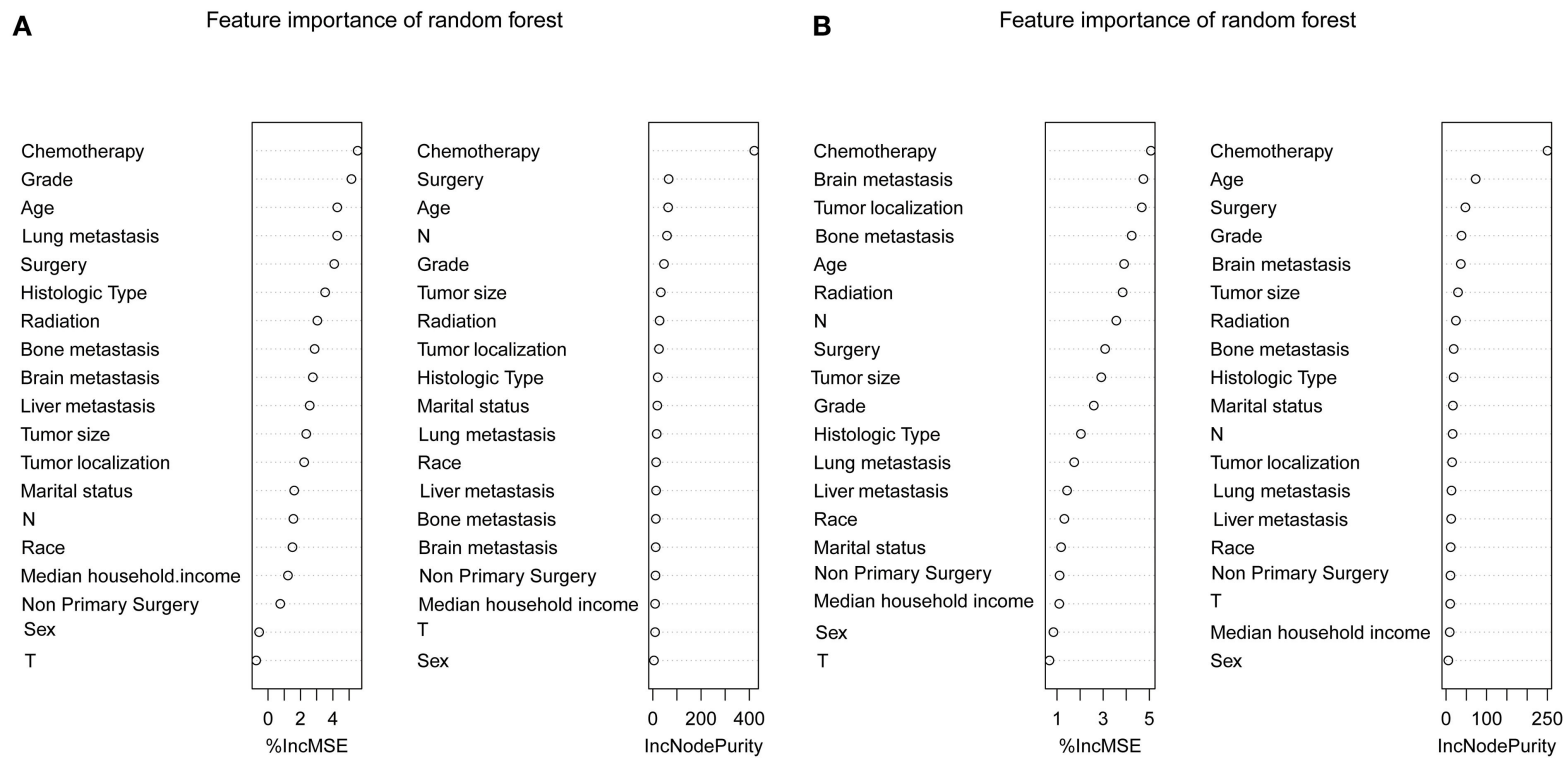
Feature importance of the random forest. Overall early death **(A)** and cancer-specific early death **(B)**.

**Figure 6:**
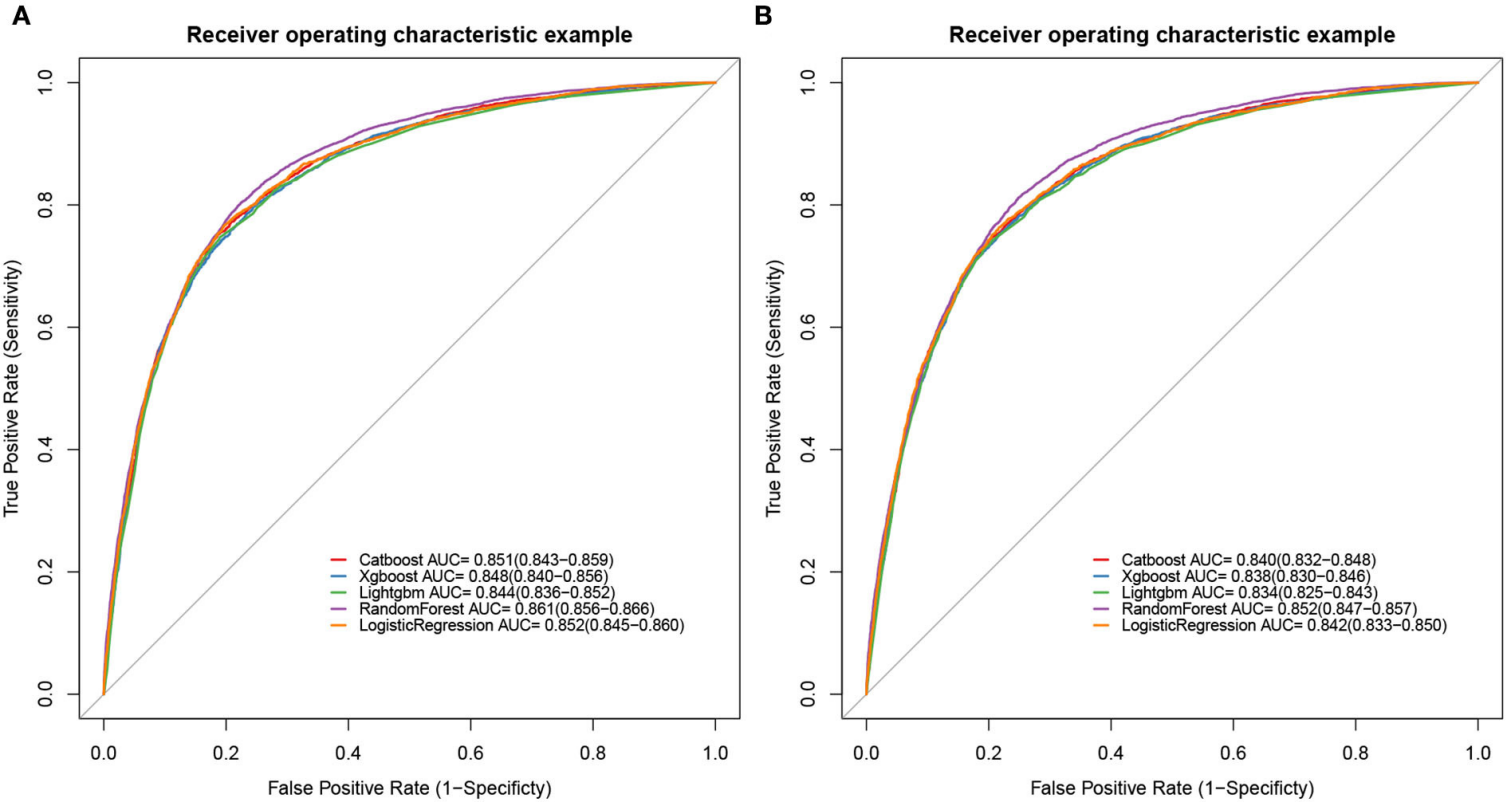
ROC curves of ML models. Overall early death **(A)** and cancer-specific early death **(B)**.

To determine the accuracy of our predictive nomogram model, novel ML algorithms were applied in the validation cohort (*n* = 10,691). The feature importance of random forest is shown in Figure 5. As shown in Figure 6, when it comes to overall early death and cancer-specific early death, the random forest model had the best performance with AUC values of 0.861 and 0.852, respectively, when compared with the XGBoost (AUC = 0.848, 0.838, respectively), LightGBM (AUC = 0.844, 0.834, respectively), CatBoost models (AUC = 0.851, 0.840, respectively), and logistic regression (AUC = 0.852, 0.842, respectively). According to the pairwise statistical comparisons between AUC values in all-cause early death (Table 4), the observed inter-individual differences in the overall performance of random forest, CatBoost, and logistic regression were statistically significantly higher than those of XGBoost and LightGBM. However, there is a slight difference in cancer-specific early death (Table 5), and the observed inter-individual differences in the overall performance of random forest were statistically significantly higher than those of logistic regression, XGBoost, CatBoost, and LightGBM. The learning rate and maximum depth used for each ML model are shown in Supplementary Table S1.

**Table 4:** *P*-values of the pairwise statistical comparisons of the ML model AUC (overall early death) values derived from the bootstrap test.

**Logistic regression**	**Catboost**	**Xgboost**	**Lightgbm**	**RandomForest**
Logistic regression	0.096	<0.001	<0.001	0.062
Catboost		0.044	<0.001	0.029
Xgboost			0.014	0.006
Lightgbm				<0.001
RandomForest				

**Table 5:** *P*-values of the pairwise statistical comparisons of the ML model AUC (cancer-specific early death) values derived from the bootstrap test.

**Logistic regression**	**Catboost**	**Xgboost**	**Lightgbm**	**RandomForest**
Logistic regression	0.118	0.006	<0.001	0.038
Catboost		0.138	<0.001	0.017
Xgboost			0.019	0.005
Lightgbm				<0.001
RandomForest				

**Figure 7:**
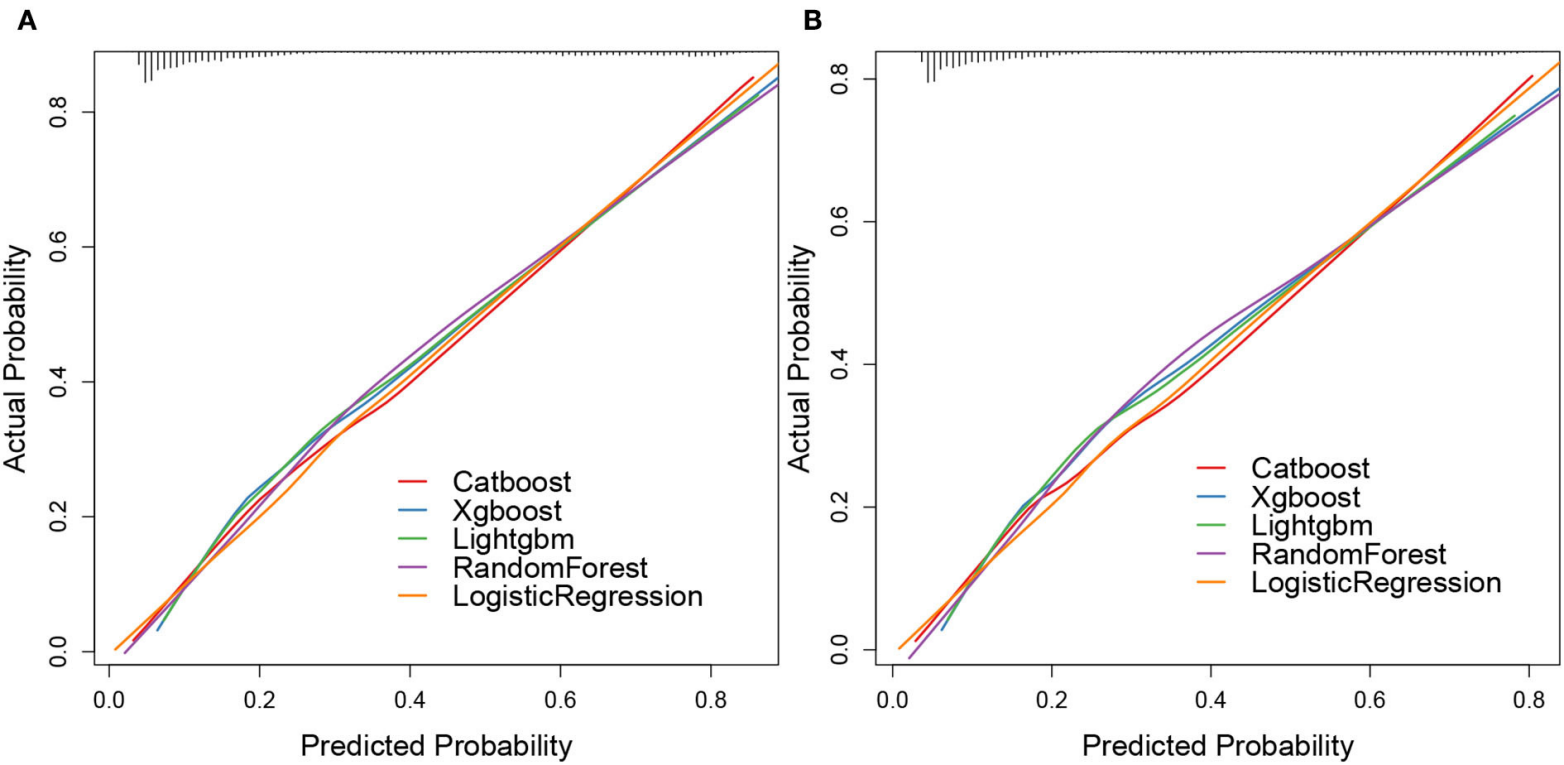
Calibration plots of ML models. Overall early death **(A)** and cancer-specific early death **(B)**.

The calibration plots of the five algorithms were subsequently constructed. We found that the lines of the validation cohort of overall early death and cancer-specific early death were all around the 45° ideal line, which showed that these algorithms had a certain predictive value. Moreover, the overall early death and cancer-specific early death in patients with mCRC predicted by the random forest model had the strongest agreement with the observed results, followed by the logistic regression, CatBoost, XGBoost, and LightGBM models (Figure 7).

**Figure 8:**
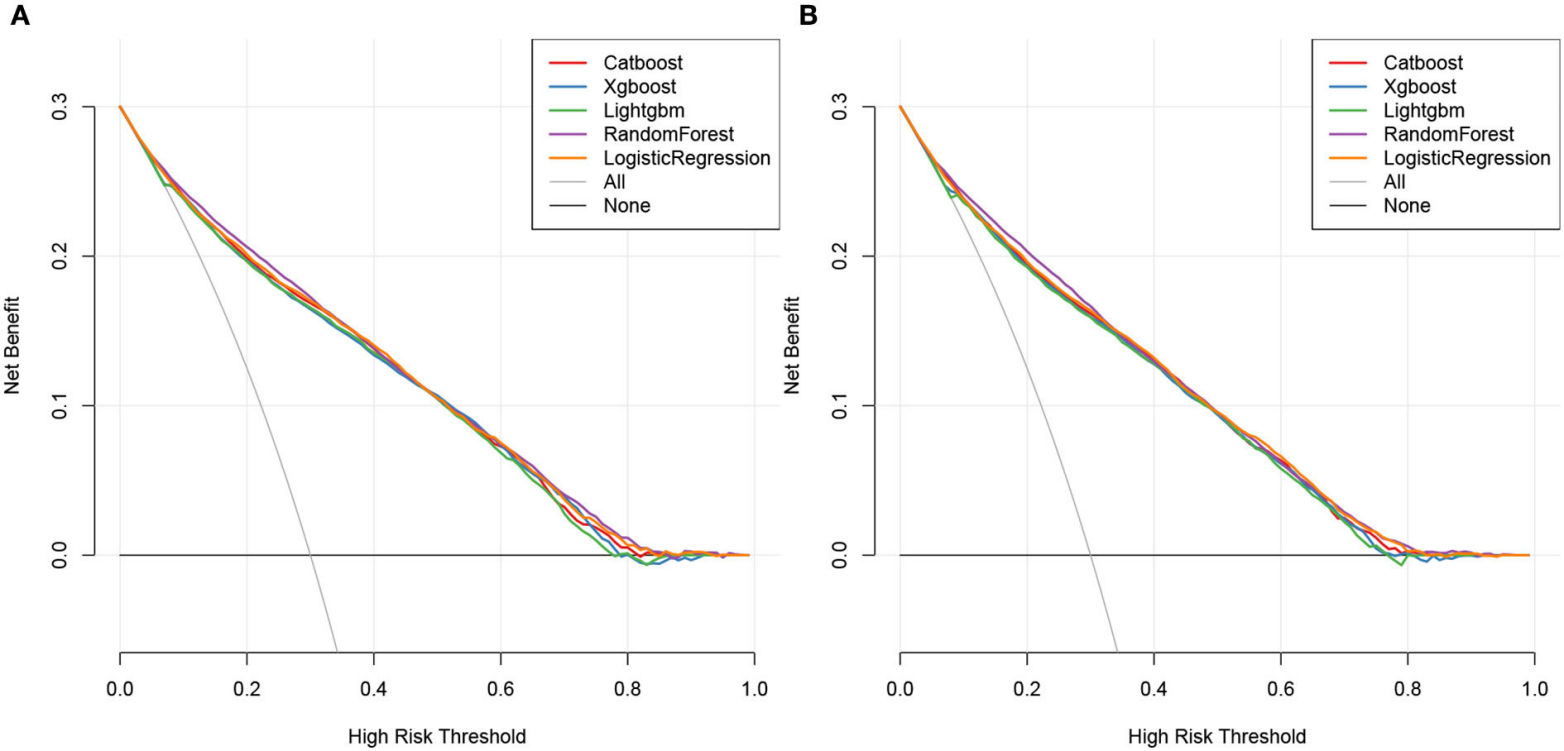
Decision curve of ML models. Overall early death **(A)** and cancer-specific early death **(B)**.

The DCA plots of overall early death and cancer-specific early death revealed that the random forest model most accurately predicted clinical outcomes, followed by the logistic regression, CatBoost, XGBoost, and LightGBM models (Figure 8).

Combining the results of ROC, calibration, and DCA curves, our study showed that the prediction models of novel ML algorithms constructed based on the aforementioned factors had higher precision and clinical applicability for predicting overall early death and cancer-specific early death in patients with mCRC than the logistic regression model.

## 4. Discussion

CRC is the fourth leading contributor to cancer-related deaths in the world. With the rapid development of treatments and the prolonged median survival time of patients with CRC (17), the early death rate is still up to 20.7%, based on the SEER database, as revealed by this research. CRC is prone to distant metastases, and lung, liver, bone, and brain are the most frequently metastatic sites (18). mCRC in more than 65% of patients recur after surgical treatment (19). The long-term survival of patients with mCRC has attracted wide public attention; however, studies focusing on early death in patients with mCRC are rare and should be conducted (20, 21).

The risk of developing mCRC rests on different factors including lifestyle, behavioral characteristics, and genetic factors. Large cohort studies have found that the consumption of alcohol and red meat, smoking, obesity, low levels of physical activity, and inflammatory bowel diseases are risk factors of mCRC (22, 23). Donnelly et al. (24) used univariate and multivariable analyses and found that old age, being unmarried, and living alone formed the independent risk factors of CRC in the United Kingdom. Moreover, Tai et al. utilized Cox analysis and found that eight factors including age, grade, surgery, and primary site were significant prognostic factors of mCRC (20), which is consistent with our research. However, compared with previous studies, our study has some distinct advantages. First, the population included in this study was larger, which makes the results more reliable. Second, this is the first research that combined nomograms and ML models to estimate the prognosis of patients with mCRC. Third, the constructed models were validated *via* a validation cohort, which makes the models more stable and reliable.

Clinically, patients with mCRC are classified according to the TNM staging system, which is recognized as the standard method for cancer staging and provides the basis for therapeutic decisions (25). However, there are some limitations in the TNM staging system. When assessing patient prognosis, it only emphasizes distant metastases, lymph node involvement, and tumor site, while other factors such as tumor size, chemotherapy, and surgery are not considered (26, 27). Therefore, in this study, nomograms and ML models were integrated with different clinical features to comprehensively estimate survival of patients with mCRC.

The entire population included our research was obtained from the SEER database, and the patients were randomly divided into the training cohort (accounting for 70%) and validation cohort (accounting for 30%). The overall early death rate of patients with mCRC was 29.2%, and the cancer-specific death rate of patients with mCRC was 26.8%. Previous research revealed that clinical and non-clinical information including age, gender, and marital status were regarded as prognostic predictors for CRC (28). In addition to these factors, our research showed that the early death in patients with mCRC was mostly associated with chemotherapy and metastatic status. Consistent with the study of Ge (29), our results showed that the overall early death in patients with left-sided mCRC is better than that in patients with right-sided mCRC, with a hazard ratio of 0.84 and a *p* < 0.001. Researchers have found that microsatellite instability and gene expression are different for different sites of colon cancer (30, 31), which may explain why CRC at different sites has different prognoses. Our results illustrated that patients with mCRC of higher grades have higher hazard ratios. As it is known, the higher the grade, the higher the degree of malignancy and the worse the prognosis. The tumor histologic differentiation grade can provide a reference for prognosis judgment and clinical treatment. Previous studies have shown that surgery significantly improves the 5-year OS of patients with mCRC (32). Tumor size is also a vital variate factor in determining the prognosis of patients with CRC. Studies have illustrated the correlation between tumor size and survival in colon cancer (26, 33), and we found that tumor size was an independent prognostic factor in patients with mCRC.

Studies focusing on early death have been applied to many advanced cancers, and it is of great importance in cancer management. Wang et al. developed a nomogram to predict the early death of patients with stage IV CRC and found that the areas under the curve were up to 75.7% (34). In Zhu et al. (14) established a nomogram model, which is an insightful method in distinguishing the early death of patients with metastatic gastric cancer. These studies have illustrated the significant predictive ability of nomograms in predicting early death in patients with cancer.

In recent years, ML has been efficient at handling multiple variables and has been widely used in cancer detection and prediction (35, 36). In this research, ML models were constructed, and ROC, calibration, and DCA were utilized to evaluate the function of the models. The results revealed that the random forest model accurately predicted outcomes, followed by logistic regression, CatBoost, XGBoost, and LightGBM models. In summary, this research analyzed the risk factors of early death in patients with mCRC and used dynamic nomograms and novel ML algorithms to construct prognostic models. The models were efficient in predicting the prognosis of patients with mCRC and can potentially help clinicians make clinical decisions and follow-up strategies.

Although the results of this study are promising, there are several limitations to this study. First, the model is based on machine learning algorithms, so the clinical interpretation of the important features screened out by the model may be difficult. Second, the model is based on the SEER database, which only contains data of North American populations, so there may be gaps in population applicability, necessitating the inclusion of broader populations in future studies. Third, this study is retrospective, and thus, prospective clinical data are needed to provide more reliable evidence for the clinical application of this study.

## 5. Conclusion

Predictive nomograms and novel ML algorithms could provide a new method for accurately predicting the early death of patients with mCRC.

## Data availability statement

The original contributions presented in the study are included in the article/Supplementary material, further inquiries can be directed to the corresponding author.

## Ethics statement

Ethical review and approval was not required for the study on human participants in accordance with the local legislation and institutional requirements. Written informed consent for participation was not required for this study in accordance with the national legislation and the institutional requirements.

## Author contributions

YZ and ZZ: conceptualization, project administration, and funding acquisition. YZ: methodology, investigation, and supervision. ZZ: software, formal analysis, data curation, and writing—original draft preparation. YZ, ZZ, and LW: validation. LW: resources. YZ and SW: manuscript—reviewing and editing. SW: visualization. All authors have read and agreed to the published version of the manuscript. All authors contributed to the article and approved the submitted version.
