# Supplementary material for: Construction and validation of nomograms combined with novel machine learning algorithms to predict early death of patients with metastatic colorectal cancer
Source: Front Public Health. 2022 Dec 20;10:1008137. doi: 10.3389/fpubh.2022.1008137 (PMC9810140; doi:10.3389/fpubh.2022.1008137)
Supplement: Supplementary file 1 [file Table_1.docx]

Table S1. The learning rate and maximum depth used for each ML model

|  | ALL-cause early death | | Cancer-specific early death | |
| --- | --- | --- | --- | --- |
| Model | Learning rate | Max depth | Learning rate | Max depth |
| RF | Not applicable | 7 | Not applicable | 7 |
| Xgboost | 0.005 | 5 | 0.006 | 4 |
| Catboost | 0.06 | 5 | 0.02 | 4 |
| Lightgbm | 0.02 | 4 | 0.02 | 4 |
| Logistic | Not applicable | Not applicable | Not applicable | Not applicable |
