# Supplementary figures and images for: Construction and validation of nomograms combined with novel machine learning algorithms to predict early death of patients with metastatic colorectal cancer
Source: Front Public Health. 2022 Dec 20;10:1008137. doi: 10.3389/fpubh.2022.1008137 (PMC9810140; doi:10.3389/fpubh.2022.1008137)

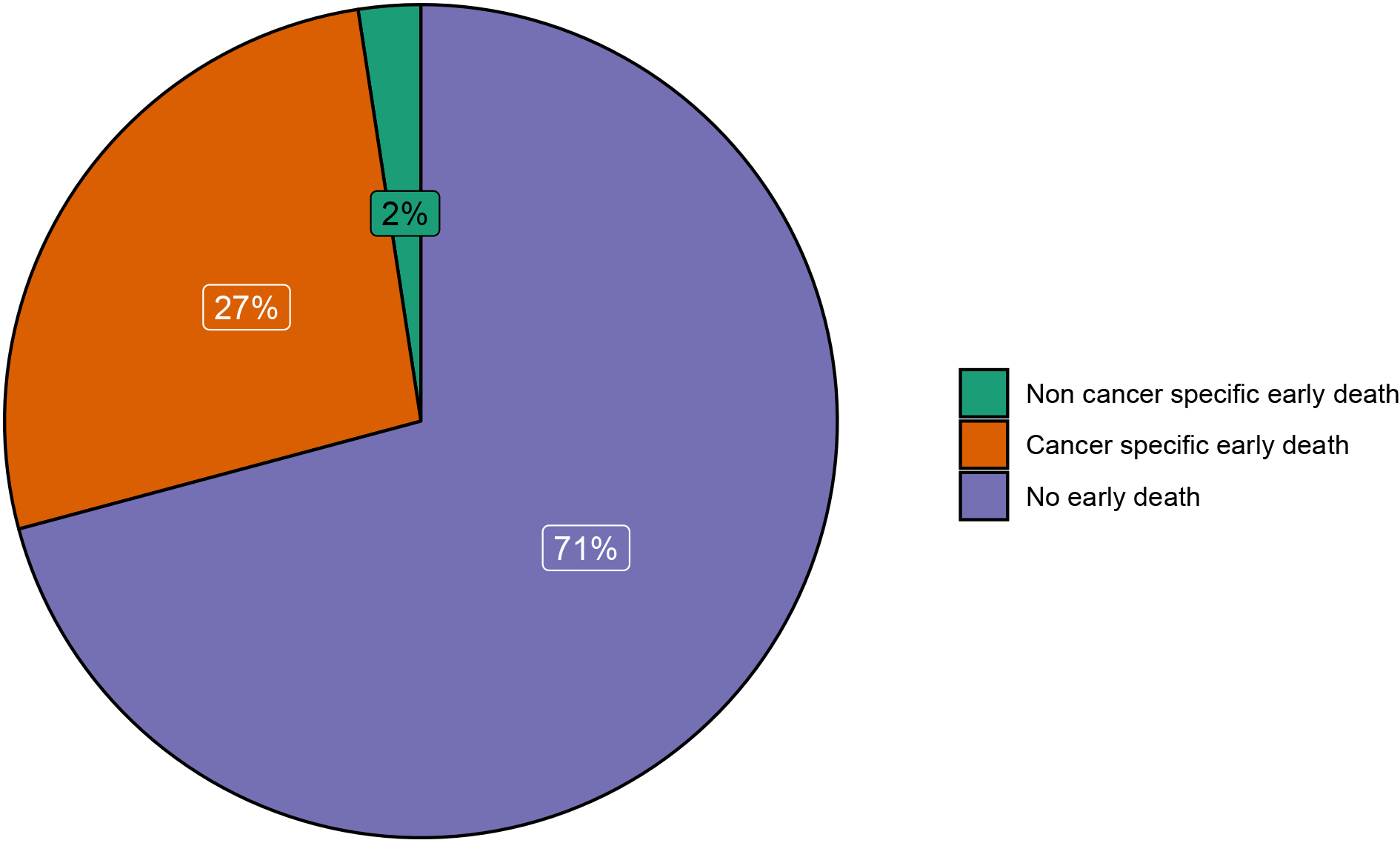

Supplement: Supplementary file 2 [file Image_1.TIF]

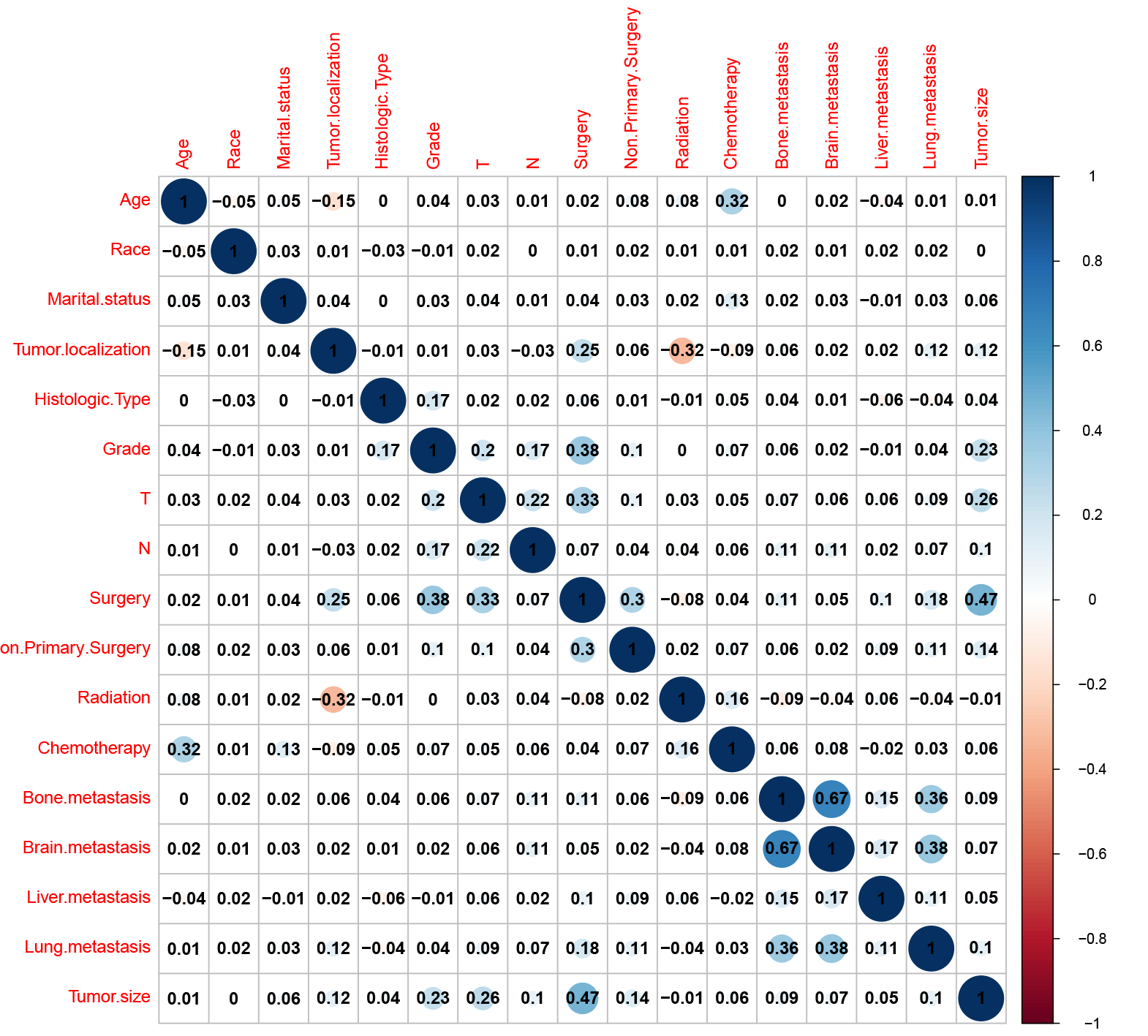

Supplement: Supplementary file 3 [file Image_2.TIF]

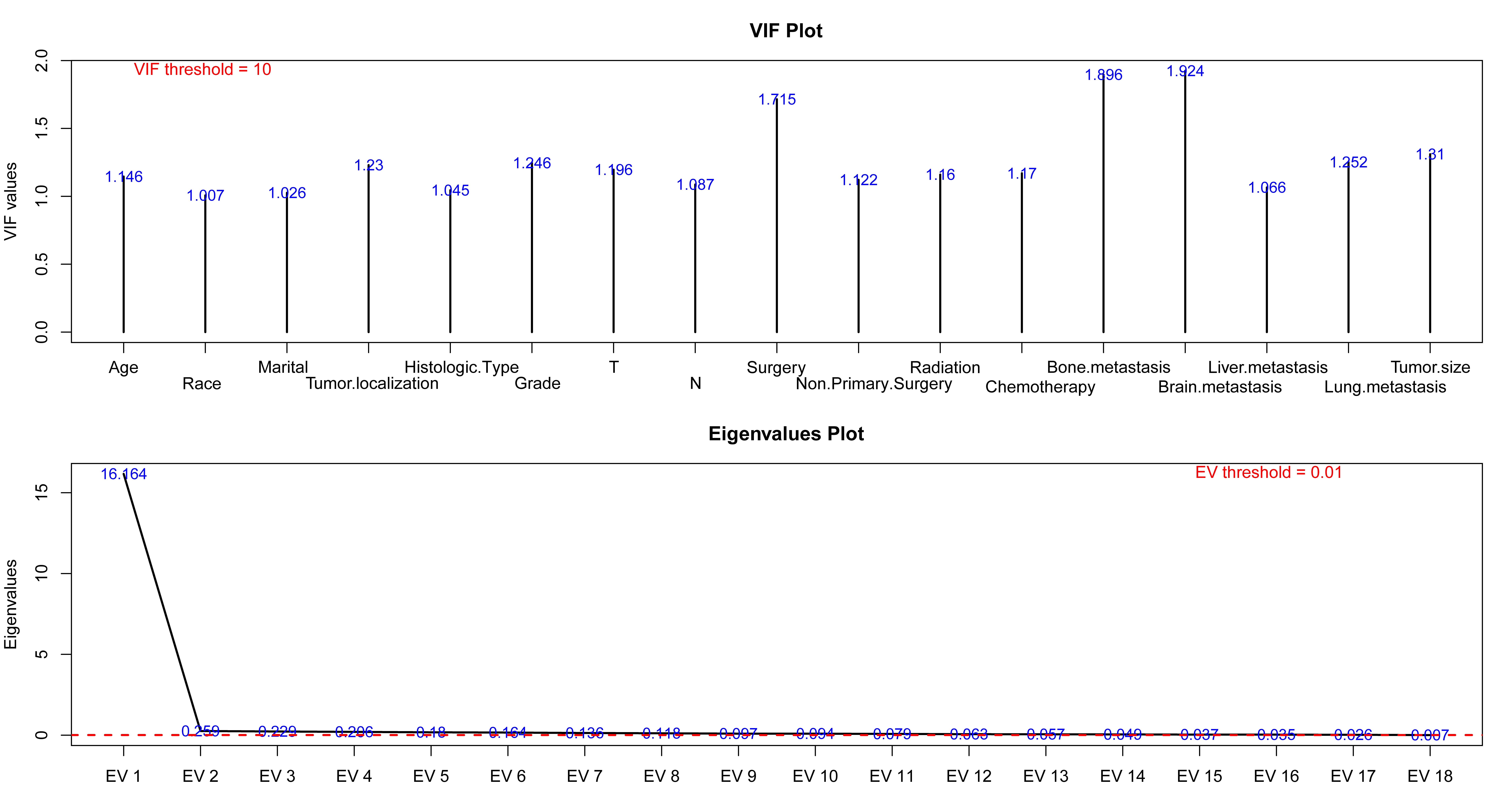

Supplement: Supplementary file 4 [file Image_3.TIF]
